# Cardiac Cancer: An Evidence-Based Study of Occurrence

**DOI:** 10.2174/011573403X349291250716112053

**Published:** 2025-07-18

**Authors:** Zahra Tolou-Ghamari

**Affiliations:** 1 Nutrition and Food Security Research Center, Deputy of Research and Technology, Isfahan University of Medical Sciences, Isfahan, Iran

**Keywords:** Frequency, tumors, cardiac cancer, SEER, SAGER, pharmacotherapy

## Abstract

**Introduction:**

From 1931 to 2025, spanning 94 years, cardiac cancer has remained a rare and sporadic tumor that poses both an investigative dilemma and a therapeutic challenge. Autopsy findings indicate that the incidence of primary cardiac malignancies is approximately 0.02 percent. Surgical resection is considered a viable and often successful treatment option.

**Aims:**

The present study aims to provide an overall assessment of cardiac cancer in Isfahan Province, Iran.

**Objectives:**

To provide detailed information on specific aspects, such as frequency and demographic characteristics of cardiac cancer.

**Methods:**

The representative data of this study were drawn from the general population of Isfahan Province. SEER (Surveillance, Epidemiology, and End Results) data were obtained from the Deputy of Health, Division of Registry of Cancer (between 2011 and 2015). With attention to subject selection (the authors followed the Sex and Gender Equity in Research (SAGER) Guidelines), and according to the ICDO topography code, C38 was considered for further investigation as heart cancer or cardiac tumors.

**Results and Discussion:**

During the study period, a total of 30,465 cancer patients were recorded, comprising 14,638 females and 15,827 males. Among these, 122 cases (0.4 percent) were identified as cardiac cancer, including 42 females and 80 males. The patients' ages ranged from 3 to 95 years, with a mean age of 46.8 ± 21.5 years. The annual distribution of reported cardiac tumors during the study period was as follows: 34, 37, 18, and 33 cases, respectively. Based on available data from the monographic code M, which does not specify subtypes, the following conditions were recorded: mesothelioma (*n* = 25), neoplasm (*n* = 11), Hodgkin lymphoma, nodular sclerosis, NOS (n = 14), and other unspecified conditions. A total of three deaths were reported.

**Conclusion:**

In the population studied, the frequency of cardiac cancer in men was significantly higher than in women. Age related to cardiac cancer in 51% was between 40-70 years old. For the patient satisfaction and financial aspects of the Iranian health system, further consideration is suggested regarding referral systems, evidence-based pharmacotherapy, and post-surgery outcome inquiries.

## 1 INTRODUCTION

Cardiac cancer is an enormously erratic form of cancer that is separated into primary and secondary tumors of the heart. Cardiac tumors can occur in any part of the heart. Historically, valvular tumors were first described by Yater in 1931, followed a few years later by Campbell and Carling, who reported a tumor on the aortic valve. In 1975, cardiac papillary fibroelastoma was identified and described by Cheitlin *et al*. [1, 2]. The infrequent cardiac tumors can be primary or metastatic [3]. The frequency of cardiac tumors has been estimated at approximately 200 cases per one million autopsies. Cardiac cancer may lead to complications such as valvular or inflow-outflow tract obstruction, thromboembolism, arrhythmias, and pericardial disorders. The diagnosis is difficult in most patients because there are non-specific symptoms such as dyspnea, night sweats, weight loss, nausea, vomiting, fever, and fatigue [4]. The rare heart cancer initiates with myxomas, fibromas, rhabdomyomas, and hamartomas [5]. The most common primary tumor of the heart is reported as a myxoma, accounting for marginally more than half of all primary cardiac tumors [3]. The recently published meta-analysis based on 74 studies investigated patients with primary (*n=*8346) and secondary (*n=*355) cardiac tumors that represented 10% of heart cancers, with the most prevalent type of benign forms. The study confirmed that the tumor subtype could be a predictor for short- and long-term mortality, and was significantly lower for benign primary cardiac tumors [6]. In the post-mortem series, the prevalence of primary malignant cardiac tumors among all primary cardiac tumors was reported to be around 25% [7]. Angiosarcoma, or cardiac sarcoma, is a malignant form of cardiac cancer that may occur in patients. Autopsies of 12487 cases in Hong Kong showed cardiac tumors in 7 cases. A study by a Vienna heart group, conducted over 15 years, showed 113 primary tumor cases, of which 11 were malignant, with a mean survival of 26.2 ± 9.8 months [8]. Primary tumors of the heart are sporadic objects whose frequency, according to surgery and autopsy reports, is 0.3% to 0.7% of all cardiac tumors. Prognosis in the young population, with malignant primary cardiac tumors, is reported to be miserable, with a survival rate of 9 to 12 months in only 10% without any surgical resection. The presentation of cardiac cancer is reported to be varied and depends on tumor location [9]. Cardiac myxoma and sarcoma (especially angiosarcomas) are reported as the most common types of benign and metastatic forms of heart cancers, respectively. According to published reports, the incidence ranges from 0.001 to 0.3% based on autopsy results [10, 11]. There are genetic tendencies regarding the cause of cardiac tumors in some patients. Autosomal disorders such as tuberous sclerosis [12] and Gorlin syndrome [13] may be linked with cardiac rhabdomyoma or fibromas [14]. A retrospective observational study conducted at a tertiary pediatric cardiac center in Iran over 8 years confirmed 41 patients with cardiac tumors out of 62975 pediatric patients referred to the center. Rhabdomyomas were the most common tumors, followed by fibromas and myxomas. The diagnosis of cardiac cancer in most cases is based on a CT or MRI scan, echocardiography, and clinical history [15].

In Isfahan, Iran, a recently published study on the prevalence and demographic characteristics of cancers reported a period prevalence of 5.7% for soft tissues [16]. Therefore, to provide detailed specific aspects such as frequency and demographic characteristics, the present study aims to focus on the overall assessment of cardiac cancer in Isfahan Province, Iran.

## 2 MATERIALS AND METHODS

In this retrospective study, all data regarding cardiac cancer (topography code C38) were obtained from the Isfahan Cancer Registry for the years between March 2011 and March 2015. The site of the cancer was documented according to the International Classification of Diseases for Oncology (Third Edition). De-identified patient names and surname data, including gender (The authors followed the Sex and Gender Equity in Research (SAGER) Guidelines), age, city of the living, date of report, topography code, dead or alive, and type of cancer, were recognized in Excel. The monography code was used to elucidate whether a neoplasm is invasive or non-invasive. For the analysis of recorded data, the Statistical Package for the Social Sciences (SPSS version 20; IBM Corp., Armonk, NY, USA) was used. Age, as a continuous variable, was expressed as mean ± standard deviation (SD). The normality of the age distribution was tested using the Kolmogorov-Smirnov test. Variables such as gender, age, alive/dead status, and year of report were expressed in terms of frequency and percentage [16-23].

## 3 RESULTS

Table **1** shows the epidemiological characteristics of patients with heart cancers in Isfahan Province, Iran. One hundred twenty-two patients with cardiac cancers corresponded to 42 females and 80 males, as shown in Fig. (**1**). With a minimum age of 3 and a maximum of 95 years, Fig. (**2**) shows the frequency of cardiac cancer according to age. The mean ± SD age was 46.8 ± 21.5 years, which was related to 40.7 ± 19.9 (minimum, 11; maximum, 83) in females and 50.0 ± 21.7 (minimum, 3; maximum, 95) in males. The distribution of age (years) among the total population studied (%) was as follows: less than 20 (11%), 20-40 (25%), 40-70 (51%), and 70-95 (13%). Fourteen patients were under the age of 20 years, corresponding to the frequency (f): 3 (f=2), 5 (f=2), 11 (f=2), 13 (f=1), 17 (f=2), 18 (f=4), and 19 (f=1) years old.

According to the available information, monographic code M was used without specifying subtypes. The following diagnostic codes were reported: code 9050 (n = 25), classified as mesothelioma (benign or malignant); code 8000 (n = 11), neoplasm (benign or malignant); code 9663 (n = 8), Hodgkin lymphoma, nodular sclerosis, NOS; code 9650 (n = 8), Hodgkin lymphoma, NOS; code 8010 (n = 5), benign epithelial tumor; and code 9702 (n = 5), mature T-cell lymphoma, NOS. Other unspecified diagnoses were also recorded.

Death data were reported in three cases, including one female (age not recorded) and two males aged 53 and 77 years. Fig. (**3**) illustrates the annual frequency of reported cardiac cancer during the study period. The number of cases recorded for each year was as follows: 34 cases in 2011–2012, 37 in 2012–2013, 18 in 2013–2014, and 33 in 2014–2015.

## 4 DISCUSSION

Abnormal tissue growth in the heart, known as cardiac tumors, represents an extremely rare form of cancer [14, 16]. The incidence of primary cardiac tumors is reported to be between 0.3 and 0.7 percent, based on surgical and autopsy data. Approximately 25 percent of primary cardiac tumors are malignant, with 75 percent of these being sarcomas. The survival rate for malignant cardiac tumors is low, with most cases resulting in death within 9 to 12 months, and an overall survival rate of approximately 10 percent [9]. Several factors may increase the risk of developing cardiac cancer, including (1) genetic predisposition, (2) a long history of cigarette smoking, (3) dysregulation of the immune system, and (4) prior exposure to radiation therapy. Although the manifestation of cardiac tumors has increased in recent years, their overall frequency remains low compared to other types of tumors [16, 24]. As a rare disease, the time interval between the detection of cardiac tumors and the subsequent diagnosis of malignancy may help clarify whether a shared or combined etiology exists [25].

It is well established that differences exist between males and females in clinical presentation, pathophysiology, disease management, and treatment response. In the present study, 34 percent of patients with cardiac tumors were female. Previous publications have reported that females accounted for 47.8 percent of 736 cases involving primary malignant cardiac tumors [26]. Another study confirmed that left atrial myxomas, the most common type of benign cardiac tumor, occur more frequently in women than in men [27]. One report indicated a female-to-male ratio of 3:1 [28], while another study found that 53 to 77.4 percent of myxoma cases occurred in female patients [29]. Specific effects of the Y chromosome have been identified in non-sex-specific tissues, including its role in protein translation within the myocardium [30]. Additionally, expression of immune-related genes on the X chromosome and differences in sex hormones have been linked to increased susceptibility to autoimmune diseases in women, which may have downstream cardiovascular consequences [31]. In this study, the mean age of the patients was 46.8 years. In addition, age was associated with <20 years old in 11% of the population studied. Previous publications reported that there are threatening signs in young and middle-aged patients regarding cardiac sarcomas that are associated with poor prognosis and overall survival ranging from 6 to 12 [32-34]. In Iran, between the years 1981 and 1993, a total of 30 cases (comprising 20 females and 10 males) with primary cardiac tumors were identified. The average age of the population studied was 32 years, ranging from 17 days to 65 years [35]. The recently published article regarding the epidemiological characteristics of different types of cancers in Isfahan, Iran, reported a period prevalence of 5.7% for soft tissues [16]. The incidence of cardiac tumors has been reported to be approximately 0.14% with the vast majority (90%) of benign type. During childhood, rhabdomyoma is the most common cardiac tumor [36].

In this study, females had a mean age of 40.7 years, which was younger than the males with a mean age of 50.7 years. Results of surgical treatment of cardiac tumors at the Münster University Hospital showed that: in 139 patients (benign; 35M/36F%) the mean age was 60.7, in 26 patients (malignant; 46M/54F%) the mean age of 54 and in 16 patients (metastasis; 63M/27F%) the mean age of 51.2 reported [37].

The 2017 meta-analysis confirmed a mean age of 68 years (including 57% females) in Japan, 48.8 years (including 45.5% females) in Austria, 50.0 years (including 45.5% females) in Spain, and 43.5 years (including 87.5% females) in France [7]. In another study, a mean patient age of 41.1 years (range, 20–63) was reported, with 44% of the patients being female [38]. In this study, there were four patients, all of whom were either 3 or 5 years old. Previous publications reported that in infants and children, the most common cardiac tumors are rhabdomyoma and fibroma [39]. Cardiac myxoma was reported as the most common heart tumor, with ages ranging from 2 to 97 years and a mean age at presentation of 50 years [40]. In agreement with previous publications confirming that benign cardiac tumors are remarkably rare but potentially deadly [41-43], in this study, three deaths were reported. The reported death in the current investigation was based on the information from the documented records of the cancer registry office obtained from the cemetery organization. Further study would be valuable to establish the underlying reasons for the correlation between mortality and cardiac tumors. Finally, giving attention to the authenticity of arrangement health system advances, mostly recommendation schemes, could be supportive in avoiding major problems in supervision equality, patient satisfaction, and economic features of the Iranian Isfahan health system.

## CONCLUSION

Cardiac tumors are uncommon but continue to be a significant part of cardio-oncology practice. Hemoptysis, breathlessness, syncope, weight loss, and sudden death are some signs associated with heart cancers. This study confirmed the frequency of cardiac tumors in Isfahan Province, Iran (0.4%). Evidence-based data confirmed the rarity of cardiac tumors in the population studied (n = 122 out of 30465 recorded patients with cancer). In 51% of cases, the age at diagnosis was between 40 and 70 years, and the frequency was significantly higher in men than in women. Although this study was limited by its retrospective nature, based on recorded data, there is a need for prospective surveys to investigate the effectiveness of combining pharmacotherapy with surgical supervision by experts in daily practice.

## Figures and Tables

**Fig. (1):**
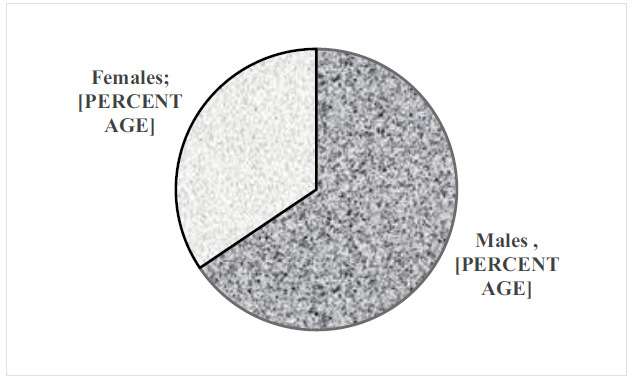
Percentage of cardiac cancer according to gender.

**Fig. (2):**
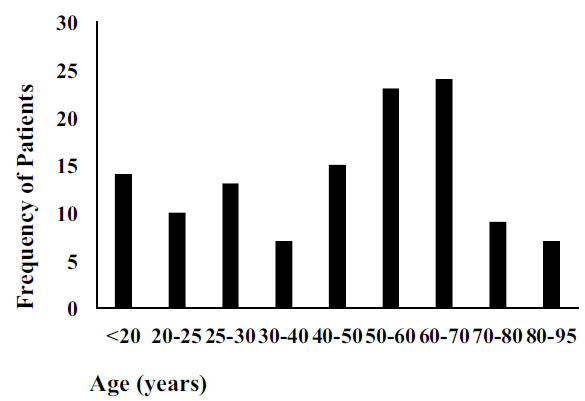
Frequency of cardiac cancer associated with different ages.

**Fig. (3):**
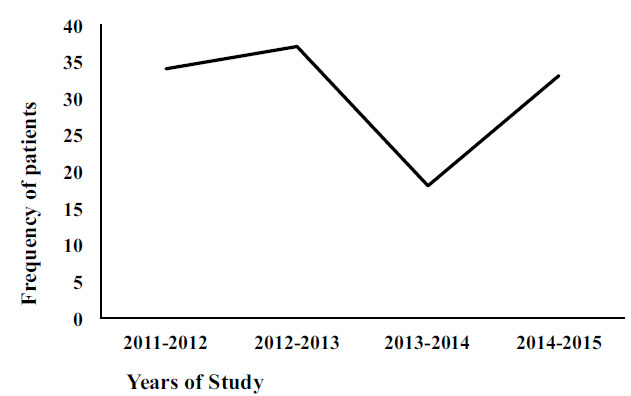
Frequency of reported cardiac cancer.

**Table 1:** Epidemiology characteristics of cardiac tumors in Isfahan Province, Iran.

**Total Cases with Cardiac Tumors**	**Gender**	**Date of Report 2011-2015**
*n=*122	Males; *n*=82 Females; *n*=40	2011-2012; *n=*34 2012-2013; *n=*37 2013-2014; *n=* 18 2014-2015; *n=* 33
Isfahan City/Rural Places	Dead/Alive	Monography Code
Isfahan City; *n*= 70 Cities around Isfahan Province; *n*=52	Dead; *n=*3 Alive; *n=*119	Mesothelioma, benign or malignant; *n=* 25 Neoplasm, benign or malignant; *n=*11 Hodgkin lymphoma, nodular sclerosis, NOS; *n=*8 Hodgkin lymphoma, NOS; *n=*6 Mature T-cell lymphoma, NOS; *n=*5 Epithelial tumor, benign; *n=*5 Epithelioid mesothelioma, benign; n=4 Others

**Note:** (*n=* Number).

## Data Availability

The data that support the findings of this study are available upon reasonable request from the corresponding author [Z.T].

## LIST OF ABBREVIATIONS

SEER: Surveillance, Epidemiology, and End Results
SAGER: Sex and Gender Equity in Research

## STANDARDS OF REPORTING

STROBE and SAGER guidelines have been followed.
